# 

**Published:** 1859-12

**Authors:** 


### TRANSLATIONS.

On Inflammation of the Cervical Lymphatic Ganglia. (*Adenite Cervicale.*) By H. Larrey, Member of the Council of Health of the Army, etc.... 749

### PROCEEDINGS OF MEDICAL SOCIETIES.

Brownsburgh Medical Society.... 750

### MISCELLANEOUS MEDICAL INTELLIGENCE.

Aneurism of the Right Carotid and Subclavian Arteries; Ligation of the Arterial Innominata. By E. S. Cooper, M.D., San Francisco.... 751

Professor Paul F. Eve.... 752

The Chicago Charitable Eye and Ear Infirmary.... 754

Illinois State Medical Society.... 755

The Smethurst Case.... 757

### BOOK AND PAMPHLET NOTICES.

A System of Surgery—Pathological, Diagnostic, Therapeutic and Operative. By Samuel Gross, M.D., Professor of Surgery in Jefferson Medical College, etc. etc. Illustrated by 936 Engravings. In two vols. Pp. 1162 and 1196. Philadelphia: Lea & Blanchard. 1859.... 760

### EDITORIAL.

Woorara as a Remedy for Traumatic Tetanus.... 761

Surgical Items:

1. Reductions of Dislocations of the Hip Joint by Manipulation.... 762
2. Exsection of five Tarsal Bones for Caries.... 763
3. Strangulated Femoral Hernia.... 763
4. Amputation at the Hip Joint.... 764
5. Resection of the Head of the Humerus for Gunshot Wound.... 764

College of Pharmacy.... 765

Rush Medical College.... 765

The "Journal".... 765

Prospectus of the Chicago Medical Journal for 1860.... 766

### RECEIPTS FOR THE JOURNAL, UP TO DECEMBER 5, 1859.

| | | | |
|---|---|---|---|
| Ill.—Dr. | John Allen, | v. 2, '59, | $2 |
| " | Thomas A. Brown, | " | 2 |
| " | W. M. Hall, | " | 2 |
| " | L. W. Carter, | balance " | 1 |
| " | A. W. Abbott, | v. 1, '58, | 2 |
| " | Graham & Manley, | v. 2, '59, | 2 |
| " | A. Mahan, | v. 1, '58, | 2 |
| " | Z. H. Madden, | " | 2 |
| " | Henrotin, | v. 1-2, '58-59, | 4 |
| " | Hitchcock, | " | 4 |
| " | L. C. Clark, | v. 2, '59, | 2 |
| " | Hiram Hall, | " | 2 |
| " | S. O. Long, | v. 3, '60, | 2 |
| " | S. York, | v. 2, '59, | 2 |
| " | Charles G. Smith, | " | 2 |
| Ill.—Dr. | H. R. Lathy, | v. 2, '59, | $2 |
| " | Francis Roualds, | " | 2 |
| " | John Wright, | " | 2 |
| " | W. W. Allport, | v. 1-2, '58-59, | 4 |
| " | L. P. Cheney, | v. 2, '59, | 2 |
| Wis.—Dr. | D. M. Marshall, | v. 1-2, '58-59, | 4 |
| " | L. C. Bricknell, | v. 2, '59, | 2 |
| " | Hays McKinley, | " | 2 |
| " | B. O. Reynolds, | v. 1-2, '58-59, | 4 |
| Ind.—Dr. | R. B. Ray, | v. 2, '59, | 2 |
| " | A. W Dewey, | v. 2-3, '59-60, | 4 |
| Mich.—Dr. | Tompkins, | v. 2, '59, | 2 |
| " | S. H. Bristol, | v. 1-2, '58-59, | 4 |
| Minn.—Dr. | Chas. Hill, | v. 1, '58, | 1 |
| Iowa.—Dr. | E. C. Kimball, | v. 2, '59, | 2 |

### INDEX TO VOL. II.

PAGE.
Aneurism Cured by Compression...............450
Atmosphere, Researches on....................623
Abstracts and Items. By W. H. Byford, M.D.........................................56, 106, 165
Annual Commencement..........................115
American Medical Association 361, 407, 190, 252, 120
American Medical Association Transactions 51
Andrews, E., M.D., on Functions of the Cerebellum. Noticed..................................104
Alkalies and Acids in Practice By E. Andrews, M. D........................................ 69
Alkaline Treatment of Rheumatism. By B. F. Schneck, M.D...................................142
Allport, W. W., D.D.S., Address to Graduates of Ohio College of Dental Surgery......... 160
Anchylosis of the Knee Joint, treated by Fracture of Femur..................................16_
Ætiology of Parenchymatous Nephritis......178
Air Passages, Foreign Body in. By C. Brackett, M.D.........................................271
Air Passages, Foreign Body in, three months. By Dr. Brainard...................................133
Appointment of Medical Professors............448
Angell, L. H., M.D., on Mortality in Aurora, Ill.....................................................471
Angell, L. H., M.D., on Expectoration of False Membrane.........................................591
Anatomy, Gray's, notice of........................498
Academy of Medical Sciences, Chicago 255, 500, 476
Avulsion of Arm and Scapula. By H. B. Horlbeck, M.D.........................................504
Attendance on Medical Lectures in London.510
Allen, J. Adams, Prof..............................580
Allen, J. Adams, Prof., Address Indroductory to Seventeenth Annual Course in Rush Medical College..............................685
Absence of the Vagina. Operations by Dr. Brainard.......................................707, 389
Amputation, Causes of Death after............609
Amputation at the Hip Joint...............191, 764
Apparatus for Extension of Flexed Knee-Joint...............................................180
Artificial Production of Bone......................288
Amputation of Shoulder Joint....................747
Aneurism of Right Carotid and Subclavian Arteries. By E. S. Cooper, M.D...............751
Amputation at the Hip Joint.......................764

Brainard, Daniel, M.D.
On Deformity from Fracture treated by a new method....................................... 5
Case of a Foreign Body in the Air Passages three months. Successful operation, and suggestions on best means of preventing hemorrhage, and obviating effects of entrance of cold air after Tracheotomy..133
Salutatory...............................................183
On the Treatment of Chronic Hydrocephalus by injections of Iodine..................197
Notes of Experiments on the Effects of Woorara introduced into the Stomach...261
Resection of the Knee Joint. Translated.285
Sequel and Ultimate Result of a Case of Resection of Knee Joint.....................340
Case of Congenital Absence of Vagina. Operation and reflections......................389
New Method of Preventing Hemorrhage in Operations on the Lips...................465
Operation for Tumor of the Throat, and on Use of Scissors which Crush the Tissues.473
Essay on Treatment of Spina Bifida by Injections of Iodine...............................517
Cases of Extirpation of the Parotid Gland and other Glandular Tumors...............709

PAGE.
Bailey, F.K., M.D., on Intestinal Concretions.150
Bailey, F. K., M.D., Letter by.....................386
Brain, Injury of. By W. T. Sherwood, M.D. 23
Baltzell, Wm. H., M.D., on Placental Presentation..................................................581
Belladonna in Preventing Secretion of Milk.647
Belladonna Poisoning. By B. F. Schneck, M.D................................................... 32
Bevan, Thos., M.D., Translations by 346, 277, 234, 227
Braun's Uremic Convulsions of Pregnancy, notice of.................................................171
Brodie, Sir Benj., Mind and Matter. notice of.170
Brower, J. H., M.D., on Cephalic Version... 11
Brown-Sequard on Temporarily Reanimating Persons Dying of Disease..................227
Bone, Artificial Production of.................288
Botany, Gray's, notice of..........................314
Byford, Wm. H., M.D., Abstracts and Items 56, 106, 165
Bishop on Scarlatina and its Treatment......242
Brackett, C., M.D., Surgical Cases.........337, 89
Brackett, C., M.D., Foreign Body in Air Passages....................................................271
Brackett, C., M.D., Cancer of Conjunctiva. Extirpation of Eye...............................158
Brackett, C., M.D., Ovarian Tumor removed by Prof. Meeker...................................337
Bird's Urinary Deposits, notice of...............499
Burn treated by White Paint, Case of.........730
Brain, Structure and Functions of............744

Clinical School suspended........................448
Cephalic Version. By J. H. Brower, M.D... 11
Cerebellum, Functions of..........................104
Correspondence.....................125, 126, 560, 122
Cook, E. P., M.D., Letter by......................122
Cancer of the Conjunctiva. By C. Brackett, M.D...................................................158
Chronic Hydrocephalus. By Dr. Brainard...197
Circulation of the Nervous Fluid...............235
Comparative Physiology. Flourens. Notice of.......................................................236
Chicago Academy of Medical Sciences 476, 500, 255
Chicago Medical Society...........................260
Convention of Medical Teachers at Louisville...............................................356, 320
Convention for Revising U.S. Pharmacopœia.323
Congenital Absence of the Vagina. By Prof. Brainard.......................................707, 389
Correction..............................................450
City Hospital....................................574, 452
Clinical Report on Pneumonia. By Prof. Austin Flint.........................................481
Carnochan's Contributions to Operative Surgery, notice of.....................................500
Cod-Liver Oil, Formulæ for Gelatinization of.507
Chloroform, Discovery of..........................508
Cream as a Substitute for Cod-Liver Oil......508
Cesarean Operation. By Borie..................570
Clinical Records......................................625
Compound Pregnancy. By M. Stahl, M.D.645
Chemistry. Fownes. Notice of ..............433
Cody, James, M.D., on Glandular Tumor of Neck........................................................658
Condie on Children, notice of....................104
Charitable Eye and Ear Infirmary......379, 754
Corpuscles of Blood, Diseases of the............738
College of Pharmacy................................765

Dysmenorrhea Cured by Hysterotome.........505
Dalton's Physiology, notice of...................103
Davis, N. S., M.D., Valedictory..................183
Diabetic Urine, Influence of Beverages......177
Deformity from Fracture, successfully treated 5

Death of Dr. Kirwin.....385
Diphtheria, Cases of. By De Laskie Miller, M.D.....539
Diphtheria. By W. G. Dyas, M.D.....661, 595
Dislocations of the Hip reduced by Manipulations.....641
Dislocated Shoulder of Six Weeks' Standing, reduced.....463
Dyas, W. G., M.D., on Diphtheria.....661, 595
Donders' Method of Assisting Vision.....466
Dickson's Elements of Medicine, notice of...559
Displacement of Coccyx.....566
Delivery by Turning.....568
Diseases of Sternum Simulating Aneurism...626
Dental Cosmos, notice of.....637

Erickson's Surgery, notice of.....103
Evil of Smoking Tobacco.....513
Epilepsy, Castration in.....498
Elephantiasis of Scrotum, Report on, notice of.....548
Extirpation of Globe of Eye for Cancer.....638
Empyema, Five Cases of. By H. Noble, M.D.652
Excision of Knee Joint.....625
Encephaloid Disease of Epididymis.....628
Enteric Fever, Practical Treatise on, notice of.632
Electricity as Anæsthetic Agent in Dentistry.512
Erectile Organs of Female, Researches on...234
Ecraseur.....510
Enemata Nutrientiæ, how absorbed..... 86
Excised Knees.....388
Eve, Paul F., Professor.....752
Exsection of Five Tarsal Bones for Caries...763

Fibrinous Concretions, Expectoration of.....591
Fatty Heart, Diagnosis of.....612
Foreign Body in Urinary Bladder.....638
Fibrin, Physiological position of.....405
Fownes' Chemistry, notice of.....433
First Announcement of Lind University.....381
Fractures, Malgaigne on, notice of.....170
Fracture of Lower Jaw..... 90
Flourens' Comparative Physiology, notice of.236
Fistula in Ano, Double.....631
Freer, J. W., M.D., Resection of Knee Joint.402

Glandular Tumor of Neck, Case of.....658
Gray's Anatomy, notice of.....498
Gray's Botany, notice of.....314
Gray's First Lessons in Botany, notice of.....314
Green on Favorite Prescriptions, notice of...318
Green, J. N., M.D., on Stomatitis Materna...137
Galt on Idiocy, notice of.....500
Gibbs, O. C., M.D., on the Treatment of Burns by White Paint.....730

Heavenridge, A., M.D., on Ligation of Artery 91
Heavenridge, A., M.D., on Pneumonia.....718
Hospital, Chicago City.....574, 452
Henry, R. F., M.D., on Obstruction of Bowels.155
Hayes, J. R., M.D., Letter by.....125
Hill, P. W., M.D., on Nostrum Venders.....126
Hypertrophy of Heart, with Hydro-Pericardium..... 20
Horlbeck on Avulsion of Arm and Scapula..504
Headland on Medicines, notice of.....674
Hancock Medical Association.....387
Hydrocephalus, Chronic, treated by Injections of Iodine.....197
Hip Joint, Amputation at.....191
Hip, Dislocation of, reduced by Manipulation.641
Holmes, E. L., M.D., on Ophthalmoscope 266, 217, 139
Holmes, E. L., M.D., on Prof. Donders' Mode of Assisting Vision.....466
Holmes, E. L., M.D., Translations by 177, 178, 180, 288
Holmes, E. L., M. D., Cases in Ophthalmic Practice.....648, 733

Illinois State Medical Society, Report of Proceedings.....438
Illinois State Medical Society.....755
Indiana State Medical Society, Annual Meeting.....442
Inversio Uteri.....341
Ingals, Prof. E., on Reduction of Dislocated Shoulder.....463
Insane Asylums, Report of.....500
Inflammation of the Cervical Lymphatic Ganglia.....749

Journal, the.....765

Kirwin, Philip, M.D., Death of.....385

Logan on Medical Education.....423
Legal Medicine, Importance of Study, notice of.....434
Legal Medicine in England and Chicago.....700
Lead, Effects of, upon Lower Animals.....112
Lead in Phthisis.....114
Lithotomy, Two Cases of.....453
Larrey on Elephantiasis of Scrotum, notice of.....548
Larrey, Father and Son.....515
Leucocytes. By Robin.....282
Lithotomy.....388
Larrey, H., on Inflammation of the Cervical Lymphatic Ganglia.....749
Ligation of the Arterial Innominata. By E. S. Cooper, M.D.....751

Macula Cachectica.....564
Mitchell's Five Essays, notice of.....319
Mutter, Prof. T. D., M.D., Death of.....191
Medical Colleges, Statistics of.....256
Medical Convention for Revising U. S. Pharmacopœia.....323
Medical Teachers, Convention of.....356, 320
Medical Association, American 407, 190, 120, 51, 361, 252
Medical Societies, Proceedings of.
Nashville.....114
Rock River Union.....502, 93
Shelby County..... 99
Champaign County.....101
Earlville Union, Annual Meeting of..... 36
Rock Island, Annual Meeting of..... 64
Chicago.....260
DeWitt County, Meeting of.....322
Hancock, Meeting of.....387
Esculapian.....435
Illinois State.....438
Indiana State.....442
Brownsburgh.....750
In Northern Iowa, Medical Convention...479
Michigan.....500
Mahla, P. F., on Compound Syrup Phosphates..... 27
Malgaigne on Fractures, notice of.....170
Mind and Matter, notice of.....170
Marshall Hall's Ready Method.....224
Mettauer on Speculum Vaginæ.....246
McArthur, A. L., M.D., on Ununited Fracture of Radius.....273
Miller, DeLaskie, Prof., on Sanitary Condition of Chicago.....325
Miller, DeLaskie, Prof., Two Cases of Diphtheria.....539
Medical Education.....423
Medical Department of Lind University.381, 572
Markoe on Subcutaneous Perforation.....424
Meigs on Woman: her Diseases and Remedies, notice of.....433
Management of Shoulders in Examining Chest, notice of.....499

Nervous Fluid Circulation....................235
Nostrum Venders....................126
Nashville Medical Society....................114
Nasal Polypus....................547, 158
Nature and Art in Cure of Disease, notice of.175
Nursing Sore Mouth....................291
New Colleges....................448
New York Colleges....................448
Nasal Carcinoma....................629
Notes of Surgical Operations....................706, 638
Noble, H., M.D., Five Cases of Empyema......652

Operative Surgery, Contributions to, notice of....................500
Opinions of Profession....................572
Obstruction of Bowels and Extra Uterine Pregnancy....................155
Ollier on Artificial Production of Bone......288
Ovarian Tumor, Operation by Prof. Meeker.337
Obituary....................643
Ovariotomy....................321
Ophthalmoscope....................266, 217, 139
Ophthalmic Practice, Cases in............648, 733

Perforation of the Femur....................747
Practical Medicine, Report on.................. 7
Phillips, G. W., M. D., on Purpura Hemorrhagica.................... 39
Poisoning by Belladonna.................... 32
Poisons. By A. S. Taylor, M.D. Notice of..241
Purpura Hemorrhagica....................564, 39
Prize Essays.................... 63
Preliminary Education....................59, 118
Physiological Position of Fibrin....................405
Pancreas, Unknown Function of....................425
Prince, D., M.D., on Lithotomy....................453
Prince, D., M.D., on Stomatitis Materna......400
Pneumonia, Clinical Report....................481
Proceedings of Medical Societies
361, 114, 93, 502, 99, 101, 435, 438, 442, 479, 476, 750
Preparatory School of Medicine....................512
Placental Presentation....................581
Procreation of Male or Female Children at will....................606
Pregnancy, Case of Compound....................645
Parrish on Pharmacy, notice of....................674
Prospectus for 1860....................708, 766
Potter, H. A., M.D., on Inversio Uteri.........341
Pregnancy and Extra Uterine Conception. By Geo. Higgins, M.D....................334
Parotid Gland, Extirpation of, Cases of......709
Pneumonia, Essay on....................718

Reynolds, B. O., M.D., on Typhoid Fever...... 30
Resignation....................122
Reeder, J. H., M.D., on Injection of Nitrate of Silver in Croup....................154
Reeder, J. H., M.D., on Tr. Muriate Ferri in Nasal Polypus....................547
Reanimating, Temporarily, Persons Dying of Disease....................227
Rouget on Erectile Organs of Female.........234
Resection of Knee Joint, Sequel....................340
Resection of Knee Joint, Freer's Case.........402
Resection of Knee Joint, Brainard's Case...707
Resection of Knee Joint, Case, translated...285
Rush Medical College......185, 115, 580, 449, 765
Referring Unread Papers....................451
Reduction of Shoulder of Six Weeks' Standing....................463
Reduction of Dislocated Hip....................641
Record of Mortality of Aurora....................471
Raw Meat in Dysentery....................489
Ræser on Displacement of Coccyx............566
Reeves on Enteric Fever, notice of...............632
Robertson, W. S., M.D., Communication......560
Reductions of Dislocations of the Hip Joint by Manipulation....................762
Resection of the Head of the Humerus for Gunshot Wound....................754

Sherwood, W. T., M.D., on Injury of Brain 23
Schneck, B. F., M.D., on Poisoning by Belladonna.................... 32
Schneck, B. F., M.D., Two Cases of Internal Injury.................... 34
Schneck, B. F., M.D., Alkaline Treatment of Rheumatism....................142
Surgical Cases. By C. Brackett, M.D......... 89
Summer Course of Medical Instruction......117
Spongy Gums....................149
Stomatitis Materna....................400, 137
Scarlatina....................410, 242
Salutatory of Dr. Brainard....................183
Speculum Vaginæ....................246
Statistics of Medical Colleges....................256
Stone, W. R., M.D., on Protracted Gestation.275
Suspension of Clinical School....................448
Stahl, M., M.D., on Compound Pregnancy...645
Straightening Limb by Brainard's Method...161
Schlœtzer, Geo., M.D., on Spontaneous Gangrene....................542
Supporting Stumps after Amputation......629
Spina Bifida....................517
Structure and Functions of the Brain.........744
Shoulder Joint, Amputation of....................747
Smethurst Case....................757
Surgery, System of, notice of....................760
Strangulated Femoral Hernia....................763

Typhoid Fever.................... 30
To our Patrons....................320
Transactions of Am. Med. Association......... 51
Taylor on Poisons, notice of....................241
Transfusion in Yellow Fever....................108
Titles of Articles in Exchanges....................258
Trephining for Epilepsy and Insanity.........639
Tobacco-Pipe Stem in Throat....................628
Tonics and Iron in Erysipelas....................625
Tumor of Parotid....................630
Tumor of the Throat....................473
Treatment of Anchylosis of Knee Joint......162
Tanner on Diseases of Infancy and Childhood, notice of....................373
Traumatic Tetanus, Woorara as a Remedy for.761

Ununited Fracture of Radius, cured by Perforation....................273
Urinary Diseases, Morland's, notice of......... 53
Utility of Permanent Exutories....................570
Urinary Deposits, Bird's, notice of............499
University of Pacific....................257
Uterine Polypus....................220
Uremic Convulsions, notice of....................171

Valedictory. By C. Goodbrake, M. D......... 41
Valedictory of Dr. N. S. Davis....................183
Variations of Color in Venous Blood......346, 277
Vesico-Vaginal Fistula....................706
Vagina, Congenital Absence of............389, 707
Ventilation, notice of....................318

Woman: her Diseases and Remedies, notice of....................433
Woorara in Stomach....................261
Woorara as a Remedy for Traumatic Tetanus....................761
Whitney Case....................575, 186
Wilson's Human Anatomy, notice of......... 52
Woodward, B., M.D., on Hypertrophy of Heart 20
Woodward, B., M.D., on Enema Nutrientiæ 86
White Corpuscles, Anatomy and Physiology of....................282
Woodworth, B. S., M. D., on Treatment of Inflammation.................... 80
Wynne, James, M.D., on Importance of Study of Legal Medicine....................434

